# RuO_2_ Nanostructure as an Efficient and Versatile Catalyst for H_2_ Photosynthesis

**DOI:** 10.1021/acsaem.3c00764

**Published:** 2023-05-24

**Authors:** Alberto Bianco, Alessandro Gradone, Vittorio Morandi, Giacomo Bergamini

**Affiliations:** † Department of Chemistry ‘‘Giacomo Ciamician’’, University of Bologna, Via Selmi, 2, Bologna 40126, Italy; ‡ CNR Institute for Microelectronics and Microsystems, Via Gobetti 101, Bologna 40129, Italy

**Keywords:** three-component system, H_2_ photogeneration in organic solvent, thiol electron−donor, commercial RuO_2_, catalyst recycling

## Abstract

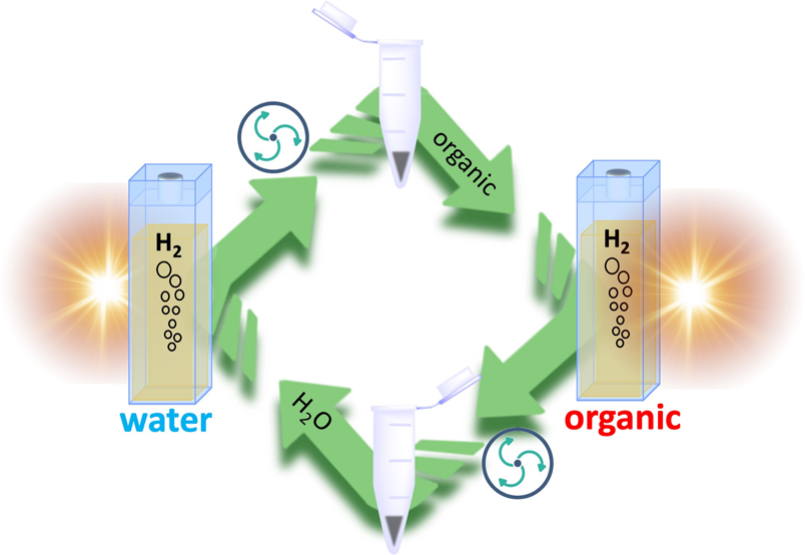

Photocatalytic H_2_ generation holds promise in the green production of alternative fuels and valuable chemicals. Seeking alternative, cost-effective, stable, and possibly reusable catalysts represents a timeless challenge for scientists working in the field. Herein, commercial RuO_2_ nanostructures were found to be a robust, versatile, and competitive catalyst in H_2_ photoproduction in several conditions. We employed it in a classic three-component system and compared its activities with those of the widely used platinum nanoparticle catalyst. We observed a hydrogen evolution rate of 0.137 mol h^–1^ g^–1^ and an apparent quantum efficiency (AQE) of 6.8% in water using EDTA as an electron donor. Moreover, the favorable employment of l-cysteine as the electron source opens possibilities precluded to other noble metal catalyst. The versatility of the system has also been demonstrated in organic media with impressive H_2_ production in acetonitrile. The robustness has been proved by the recovery of the catalyst by centrifugation and reusage alternatively in different media.

## 1 Introduction

The international community is actively promoting the development of clean and sustainable energy sources to fight against the energy, environmental, and economic crises arising from the severe dependence on burning conventional fossil fuels.

Hydrogen (H_2_) is the most promising candidate as a fuel of the future because it has the highest gravimetric energy density (120 MJ kg^–1^)^1^ and water is the sole “waste” product. Thus, the development of an efficient, stable, and sustainable system for green H_2_ production is deemed as the “Holy Grail” of energy conversion.^2,3^ Molecular hydrogen is considered so fundamental in energy transition that all the possible sources, conditions, and ways to produce it are coveted.

Harvesting and converting solar energy, which is clean, inexpensive, very abundant, and equally distributed around the globe, into H_2_ could be the best strategy to face all these challenges at once.^4^ The research in this field relies mainly on three different approaches, namely, electrolysis powered by photovoltaic panels (PV + EC), photoelectrochemical cells (PEC), and photocatalysis (PC). The latter is composed by three different steps: the absorption of light and the subsequent charge generation, the spatial separation of these charges, and the hydrogen evolution reaction (HER). H_2_ generation, since it is a multielectron process, is boosted by a hydrogen evolution catalyst (HEC), which can be either a molecule or a material.^2,5−10^ Among the plethora of HECs, the most widely used are platinum and Pt-based catalysts,^7,11−13^ which are very efficient, but, due to its high cost, low availability, and tendency to be poisoned by several compounds, their implementation remains extremely challenging.^14^ Other noble metals demonstrated activity in HER,^11,14^ and among them, ruthenium, which is at least 6 times cheaper than Pt,^15^ has already been employed in 1979,^16^ but it gained greater attention only in the last few years,^17^ showing HER overpotential at 10 mA cm^–2^ very close to that of Pt.^18,19^ Moreover, the oxides of Pt group metals are widely used as an oxygen evolution catalyst (OEC),^20−22^ but some of them also showed good activity in HER.^23^ In particular, ruthenium (IV) oxide (RuO_2_) has been extensively studied as an OEC,^24−28^ scarcely for H_2_ evolution, and mostly as an electro-^23,29−33^ or a photoelectro-HEC,^34−37^ but it is growing as demonstrated by the increasing number of papers since the last 10 years (Figure S1). The RuO_2_-based electrodes and nanoparticles (Nps) are exploited as a HEC by applying an external bias to induce metal reduction and thus favoring the formation of Ru–H bonds and consequent H_2_ evolution.^15,38^ Several examples in which Ru or RuO_2_ Nps are supported on other metal oxides and employed as colloidal dispersion for H_2_ or O_2_ evolution demonstrated improved activity, stability, and recoverability.^15,17,27,39,40^

**Figure 1:**
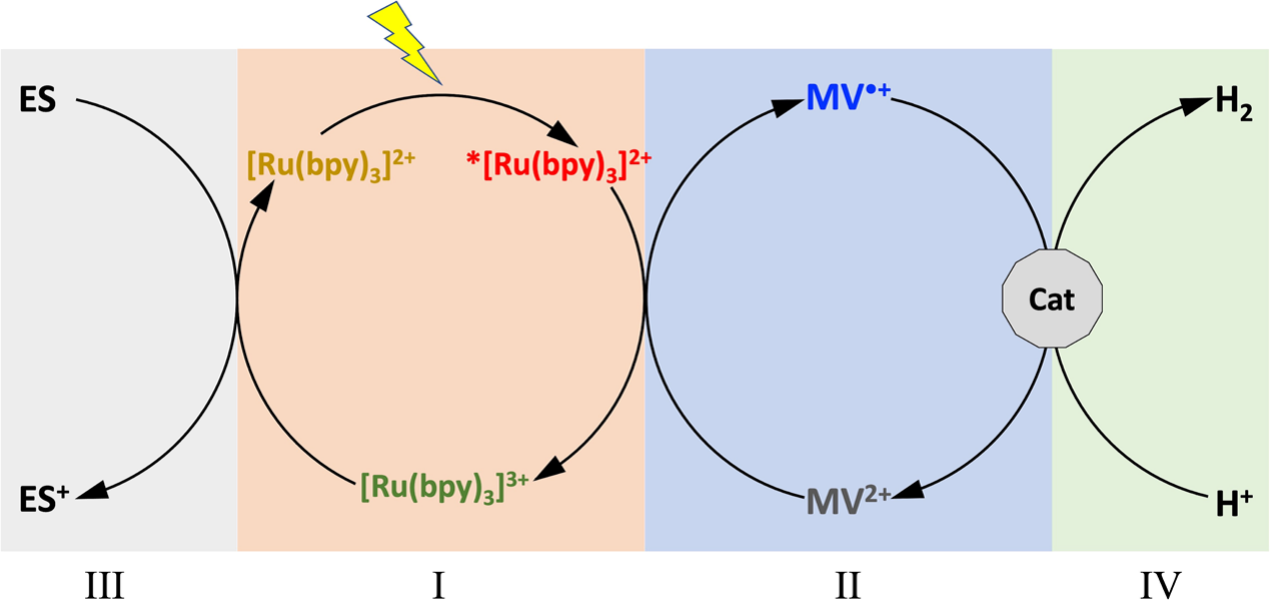
Schematic representation of the photocatalytic steps.

Here, we proposed the combination of a well-known photoinduced electron-transfer homogeneous system with commercial RuO_2_ powder for efficient H_2_ photosynthesis. We used the so-called “three-component system”^5^ approach in which a one-electron photosensitizer, a redox mediator, and a redox-storing catalyst, with the addition of a sacrificial agent/electron source, are able to convert a one-electron excited state in a two-electron proton reduction (Figure 1). The photosensitizer, ruthenium tris-bipyridyl ([Ru(bpy)_3_]^2+^), once excited, transfers one electron to methyl viologen (MV^2+^) generating [Ru(bpy)_3_]^3+^ and MV^•+^ (I and II in Figure 1), and an electron source (ES) restores the starting [Ru(bpy)_3_]^2+^ (III in Figure 1), giving the possibility to accumulate the reduced methyl viologen. In the seminal papers by Grätlzel^41^ and Kagan,^42^ the ES was either an aliphatic amine or ethylenediaminetetraacetic acid disodium salt (EDTA·2Na), and, through the use of platinum Nps (PtNps) as a catalyst, they demonstrated the evolution of H_2_ in water (IV in Figure 1).^41−43^ Despite the great number of studies on photocatalytic generation of molecular hydrogen, this system, coming from the seventies, remains one of the most simple, stable, and efficient. Two of the main drawbacks of this approach are related to the use of platinum as a HEC because, concurrently with H_2_ evolution, (i) it is able to hydrogenate the reduced mediator^44^ and (ii) due to its poisoning restricts the choice of a compatible ES. This urges the researcher to find alternative materials, possibly cheaper, more stable, and more selective, to make a step further in H_2_ photoproduction. In this paper, we replaced the PtNps with commercial RuO_2_, and we tested its catalytic activity in different experimental conditions including those in which Pt is inactive. The aim is to use visible light, instead of an external voltage, to generate a reducing environment (45) that is able to reduce the surface of the RuO_2_ Ns and, therefore, to promote H_2_ generation. The mechanism proposed for steps II–IV (Figure 1) is the creation of Ru^0^ sites at the surface of the RuO_2_ Ns, in which the binding and reduction of H atoms take place.^46^

## 2 Results and Discussion

Commercial RuO_2_ (anhydrous, >99.9%) was treated with a top-down approach through ball-milling at 150 rpm for 30 min in an agate jar; the resulting powder was then added in the selected solvent (water, acetonitrile, dimethylformamide, or dimethyl sulfoxide) to obtain a black dispersion (0.1% m/V). 1 mL of the latter was centrifugated at 1000 rpm (90 G) for 10 min, getting as a supernatant a dark gray dispersion of RuO_2_ nanostructures (Ns). Quantification of the catalyst was done after this step, removing the supernatant and weighing the precipitate, obtaining 0.60 mg of pellet, and so obtaining the concentration of RuO_2_ in the supernatant of 0.04% m/V.

The scanning transmission electron microscopy (STEM) micrographs show that the system appears to be composed by Np aggregates, with an average particle dimension of less than 3 nm in diameter (Figure S2). The dynamic light scattering (DLS) size distribution of RuO_2_ Ns in water yields an average hydrodynamic diameter of 160 nm with a polydispersity index of 0.06 (Figure S3). In accordance with STEM observations, this value refers to the Np aggregates.

To perform rapid pre-screening of the so-prepared catalyst in different experimental conditions, we employed a 3D printed gastight cell holder equipped with a H_2_ sensor based on an Arduino microcontroller (see the Supporting Information for details).

### 2.1 pH and Loading Effects on H_2_ Photogeneration

**Figure 2:**
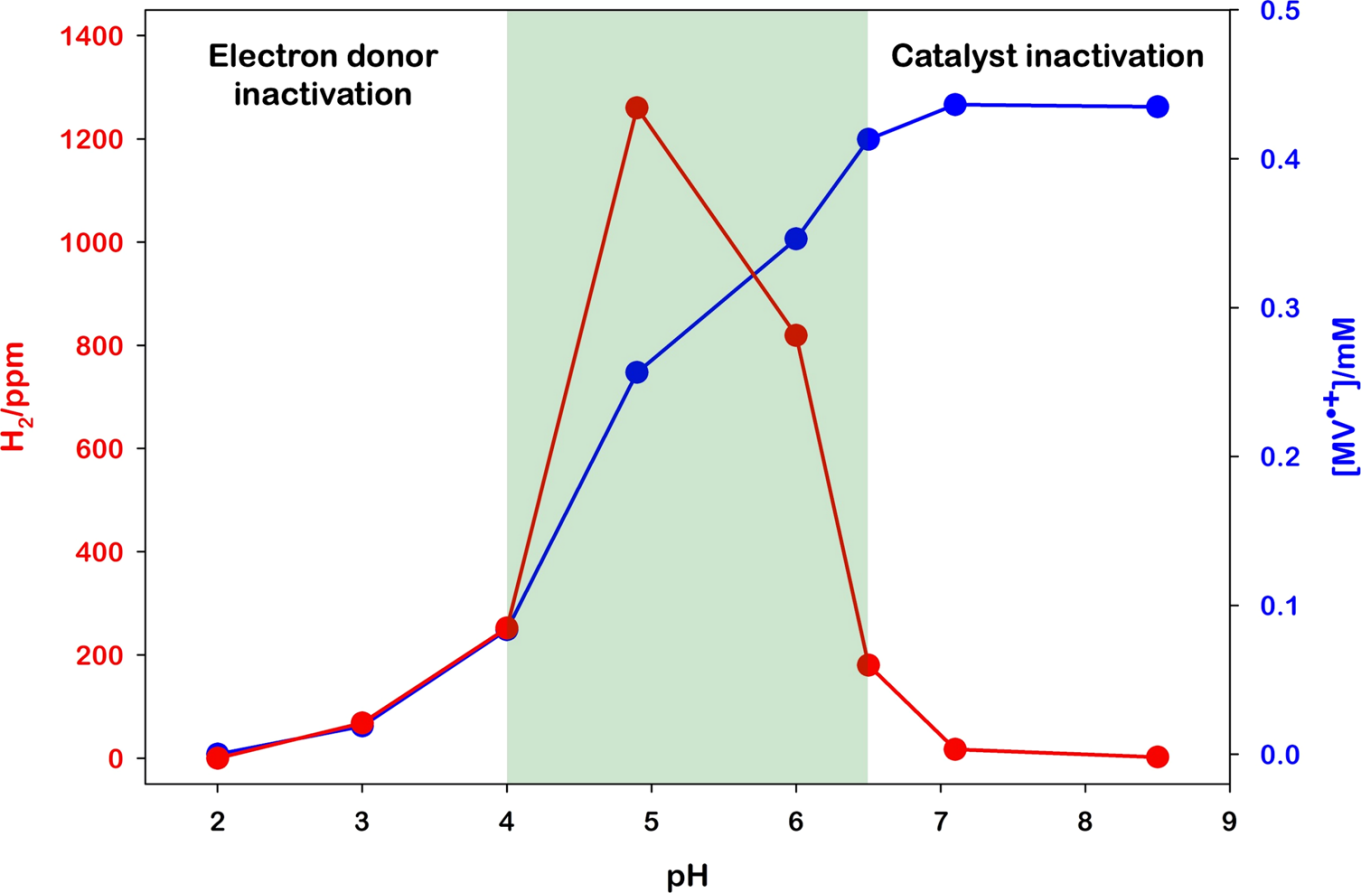
Comparison of the photoaccumulated MV^•+^ (without RuO_2_, blue line) and the photoproduced H_2_ (with 0.1 mg RuO_2_, red line) obtained upon 120 s irradiation at 460 nm of 2 mL [Ru(bpy)_3_]^2+^ (25.0 μM), MV^2+^ (5.0 mM), and EDTA·2Na (0.1 M) at different pH values.

First of all, we measured the activities of RuO_2_ Ns in photocatalytic generation of H_2_ at different pHs (Figure 2), keeping constant the other players. For these experiments, 2 mL of aqueous solutions composed of [Ru(bpy)_3_]^2+^ (25.0 μM), MV^2+^ (5.0 mM), EDTA·2Na (0.1 M), and RuO_2_ (0.04 mg) was adjusted to different pH values using 6 M HCl or 6 M NaOH and irradiated under vigorous stirring with a monochromatic light (460 nm high-power LED, see the Supporting Information for spectral irradiance) monitoring the H_2_ evolution with the Arduino sensor. The red curve in Figure 2 represents the H_2_ production spanning the pH from 2 to 8.5. We observed a maximum around pH 4.9 and a decrease in the activity at basic and acidic conditions. To rationalize the decrease of the catalytic activity at low and high pH, we compare the formation of MV^•+^ photoaccumulated in the absence of RuO_2_ (sectors I, II, and III in Figure 1, determined by the absorption spectrum) and so, by exclusion, figure out the rate-determining step of the process.

We observed that in acidic conditions, where normally the H_2_ formation is favored, no MV^•+^ is produced, whereas, raising the pH, an increasing amount of MV^•+^ is formed. This behavior can be ascribed to the protonation of EDTA that leads to a lack of chemical reduction of [Ru(bpy)_3_]^3+^ (III–I, Figure 1) and, as a result, to the back-electron transfer from the reduced viologen to the oxidized Ru complex. On the other hand, the decrease of H_2_ generation at basic pH is imputed to an increase of the 2H^+^ → H_2_ overpotential which prevents the catalytic activity of RuO_2_.^38^ With the present partners, the range of best activity is identified between pH 4 and 6.5, but presumably, using a suitable electron source at low pH, the HEC operates all the range below pH 6.5.

**Figure 3:**
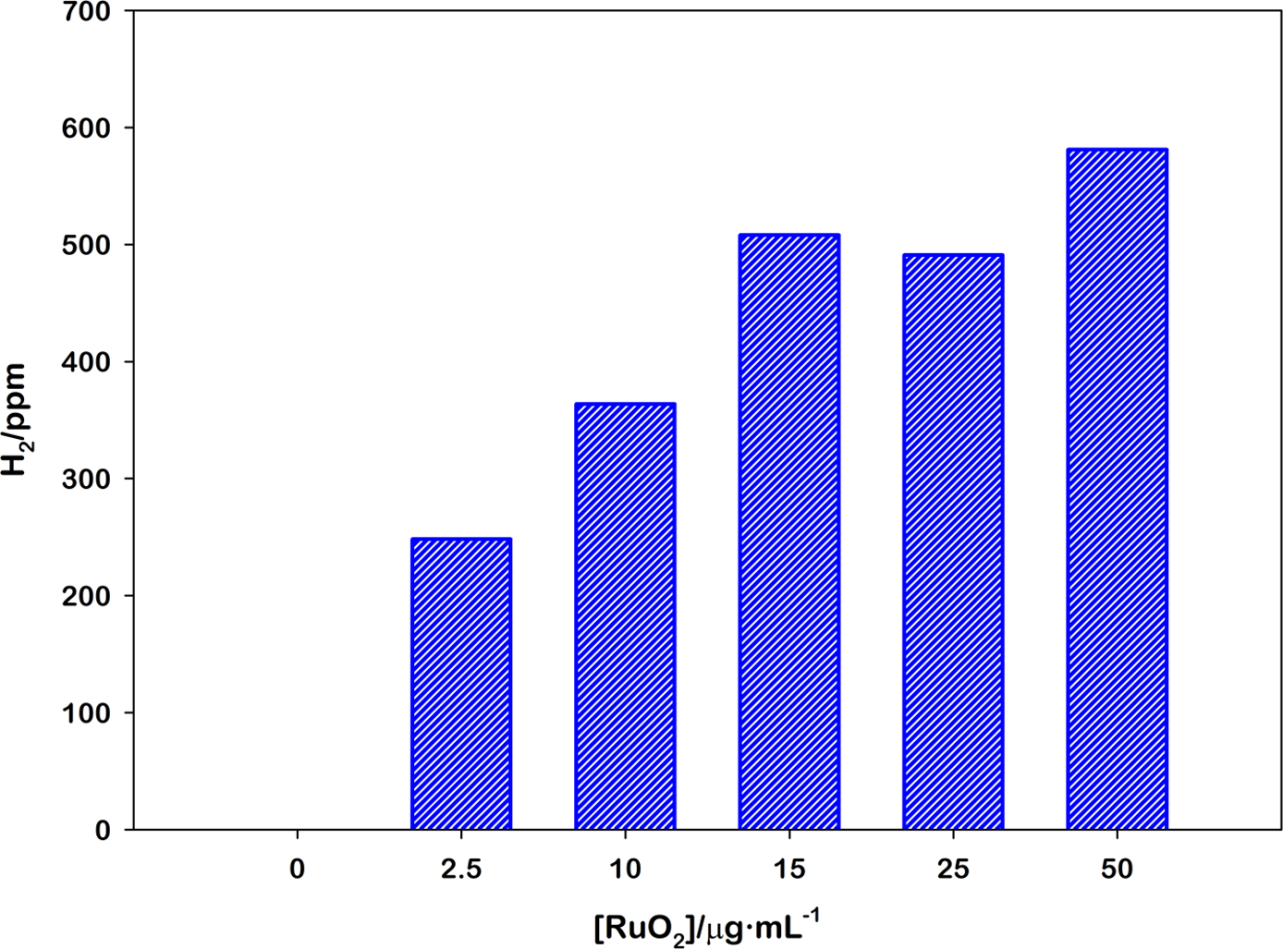
Photoproduced H_2_ for different RuO_2_ loadings obtained upon 60 s irradiation at 460 nm of 2 mL [Ru(bpy)_3_]^2+^ (25.0 μM), MV^2+^ (5.0 mM), and EDTA·2Na (0.1 M) water solution at pH 4.9 in the Arduino sensor.

Once the optimal pH value is determined, we carried out photoirradiations in the same experimental condition and varying the RuO_2_ loadings from 2.5 to 50 μg mL^–1^ (Figure 3). As expected, we observed a lessening of the activity decreasing the amount of RuO_2_ but with a very good H_2_ production already at 15 μg mL^–1^.

### 2.2 H_2_ Photoproduction in Water

After the identification of the best experimental conditions, we have moved to a more accurate measurement of the H_2_ evolution rate in continuous flow with a gas chromatograph (see the Supporting Information for instrumental details and calibration); in the same measurement, an eventual concurrent CO_2_ evolution is also detected. The experimental setup is composed of a cylindrical quartz cell (50 mm pathlength) tightly connected to the gas chromatograph in which we irradiate 10 mL of [Ru(bpy)_3_]^2+^ (30.0 μM), MV^2+^ (5.0 mM), and EDTA·2Na (0.1 M) water solution at pH 4.9 (solution as prepared, without any further adjustment) to which is added 0.20 mg of centrifugated RuO_2_.

**Figure 4:**
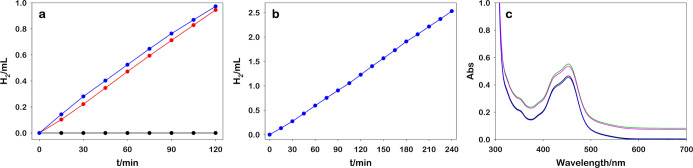
(a) Photoproduced H_2_ obtained upon irradiation at 460 nm of 10 mL [Ru(bpy)_3_]^2+^ (30.0 μM), MV^2+^ (5.0 mM), and EDTA·2Na (0.1 M) water solution at pH 4.9 using as a HEC 0.20 mg of RuO_2_ (blue line), 0.13 mg of PtNps@PVA (red line), and no catalyst (black line); (b) photoproduced H_2_ upon prolonged irradiation at 460 nm of [Ru(bpy)_3_]^2+^ (30.0 μM), MV^2+^ (5.0 mM), EDTA·2Na (0.1 M), and RuO_2_ (0.20 mg) water solution at pH 4.9; and (c) absorption spectra of the solution before RuO_2_ addition (black line), with RuO_2_ (green line), after irradiation (pink line), and after subsequent centrifugation (blue line).

For comparison, we performed the same experiment employing polyvinyl alcohol (PVA)-coated platinum Nps (PtNps@PVA, see the Supporting Information for synthesis and characterization), known as one of the most efficient HEC,^47^ in the same metal molar amount of RuO_2_ (see the Supporting Information for calculation). In Figure 4a, the results for RuO_2_, PtNps@PVA, and a control experiment without the catalyst are reported.

In order to perform a comparative assessment of the H_2_ photoproduction, we calculated a hydrogen evolution rate of 0.137 mol h^–1^ g^–1^ (in g the mass of the catalyst), similar to Pt and RuO_2_ (see Table S1 for the summary of the results), and, by measuring the incident photons at the surface of the photoreactor, we computed an apparent quantum efficiency (AQE, see the Supporting Information for details and Table S2 for the summary of the results) of 6.8%. This number is clearly affected by the nature of the photoinduced processes (I–II in Figure 1), since the electron transfer relies on the dynamic collision of *[Ru(bpy)_3_]^2+^ and MV^2+^ that, in these experimental conditions, leads to a quenching of the 50% of the excited states (Figure S5).

One of the main drawbacks of photochemical energy conversion, in particular, in the presence of molecular units, is the stability of the system which normally suffers from component degradation. We proved the stability of the catalyst irradiating the system for 4 h with no significant changes on the H_2_ evolution rate and no degradation of the photosensitizer (measured by absorption spectroscopy, Figure 4b,c). Another limitation in the employment of metal Nps, stabilized or not, as a HEC in a homogeneous solution is the challenging recovery of the catalyst due to aggregation, precipitation, surface passivation, and other inactivation processes. In this direction, we performed the recycling of the RuO_2_ catalyst after the photocatalytic cycles by centrifugation and re-dissolving, and we obtain the same catalytic activity after 5 cycles (see the Supporting Information for a detailed procedure). STEM micrographs recorded before and after the photoirradiation demonstrated the retaining of RuO_2_ morphology and composition (Figure S2).

Exploring the compatibility of RuO_2_ Ns with another electron source, we replaced EDTA·2Na with l-cysteine, a natural amino acid which is able to reduce the oxidized [Ru(bpy)_3_]^3+^ complex (III–I Figure 1)^41^ but normally not employed in hydrogen evolution since noble metal catalysts are impeded by thiols units.^48^

**Figure 5:**
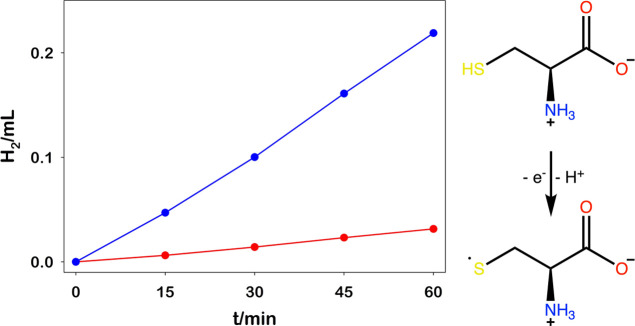
Left: photoproduced H_2_ obtained upon irradiation at 460 nm of [Ru(bpy)_3_]^2+^ (30.0 μM), MV^2+^ (5.0 mM), and l-cysteine (0.1 M) water solution at pH 4.9 using as a HEC RuO_2_ (blue line) and PtNPs@PVA (red line); right: monoelectronic l-cysteine oxidation mechanism.

As reported in Figure 5, RuO_2_ showed an excellent activity in HER using this natural amino acid as an electron source. Employing thiols as a source of electrons opens possibilities, practically unexplored because of the incompatibility of the metallic Np catalyst (e.g., Pt Nps) and surface coordinating thiol-based molecules, to combine the synthesis of value-added sulfide-based products along with H_2_ evolution.^49^ The formation of l-cystine as the oxidation product of l-cysteine is confirmed by infrared spectroscopy (see the Supporting Information, S10). Moreover, as demonstrated by the gas chromatography (GC) measurements (Figure S8), the use of l-cysteine avoids the simultaneous evolution of undesired carbon dioxide as it happens with EDTA.^50^ This aspect is not irrelevant because most of the oxidation processes usually coupled to hydrogen photoevolution generate CO_2_ as the final product, therefore getting a “dirty green” H_2_.^51^

### 2.3 H_2_ Photoproduction in Organic Solvents

**Figure 6:**
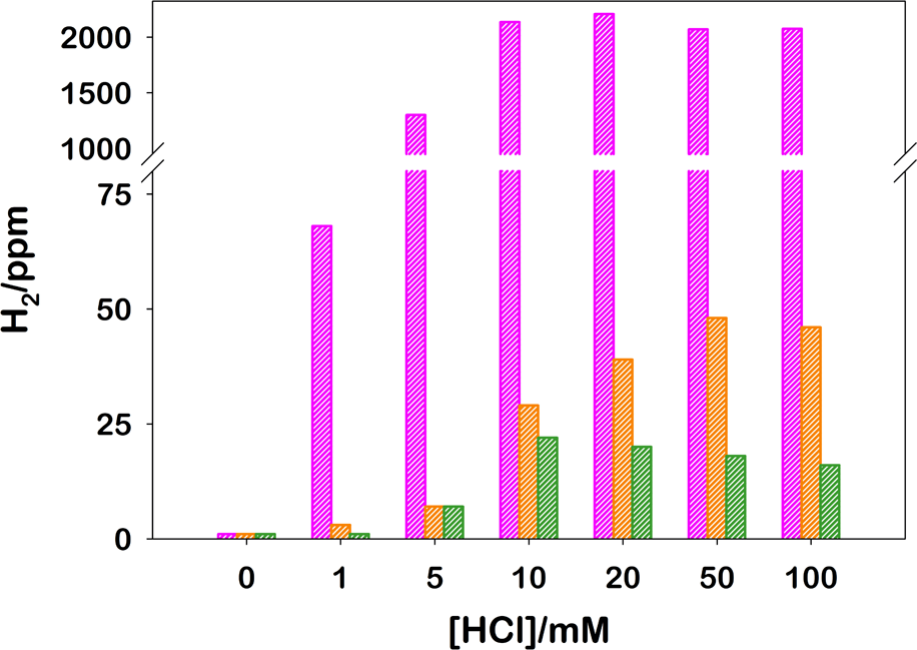
Photoproduced H_2_ obtained upon 180 s irradiation at 460 nm of 2 mL [Ru(bpy)_3_]^2+^ (25.0 μM), MV^2+^ (5.0 mM), TPP (0.1 M), and RuO_2_ (0.04 mg) in acetonitrile (pink), dimethylformamide (orange), and dimethyl sulfoxide (green) at different HCl concentrations in the Arduino sensor.

Given the versatility and robustness of RuO_2_ as a HEC, we expanded the exploitation of this catalyst in organic media to open the feasibility of using species (in particular electron sources) not soluble in water. To test the performances of RuO_2_ in organic media, we employed the same three-component system using triphenylphosphine (TPP) as the electron source.^52^ Since in an organic environment the availability of protons is limited, we adjusted the proton concentration by adding HCl in different amounts in order to maximize H_2_ production. We selected acetonitrile, dimethylformamide, and dimethyl sulfoxide as a solvent for the compatibility with the photoactive components, but nothing prevents the employment of the catalyst in other organic media. In Figure 6, using the Arduino sensor, we compared H_2_ produced in the different solvents and at different HCl concentrations. It is evident that (i) the photocatalytic cycle needs a proton source, (ii) the acetonitrile is the best solvent for this system among those tested, and (iii) increasing the amount of acid up to 10 mM results in the increase of the photoproduced H_2_, after which no further increase on hydrogen evolution rate is observed.

**Figure 7:**
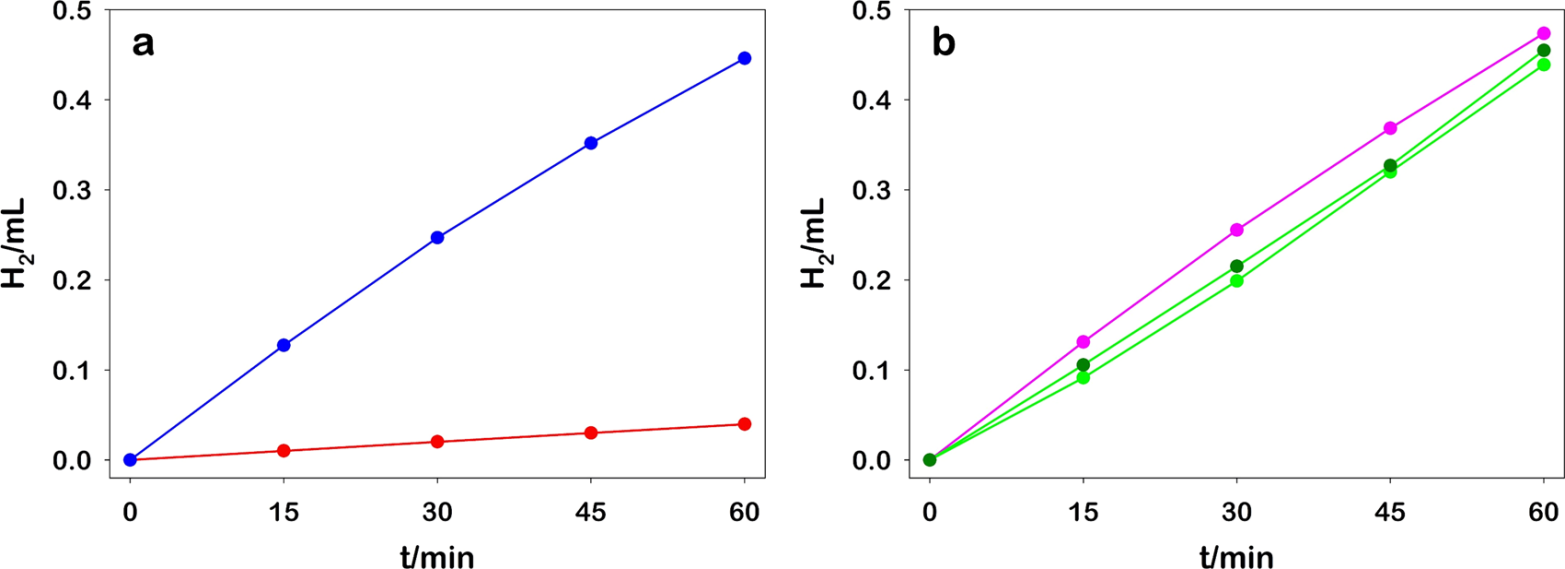
(a) Photoproduced H_2_ obtained upon irradiation at 460 nm of 10 mL [Ru(bpy)_3_]^2+^ (30.0 μM), MV^2+^ (5.0 mM), TPP (0.1 M), and HCl (10.0 mM) acetonitrile solution using RuO_2_ (blue line) and PtNPs@PVA (red line) and b) photoproduced H_2_ obtained by recycling the same RuO_2_ as a HEC in water (first cycle, green line), in acetonitrile (second cycle, pink line), and in water again (third cycle, green line dark dots). The irradiation conditions were the same as before.

The exact rates of the H_2_ photosynthesized in acetonitrile have been estimated with the GC setup. Figure 7a reports the comparison between the activities of RuO_2_ and PtNps@PVA. The production rate of H_2_ obtained using RuO_2_ in acetonitrile is comparable to that in water, whereas Pt confirms very poor activity in this media.

### 2.4 RuO_2_ Recycling

As demonstrated in water, the RuO_2_ Ns can be recovered by centrifugation also from the organic media. We thus carried out a sequence of utilization and recycling of the same HEC sample. We performed a first photocatalytic experiment in water, in the same configuration as in Figure 4b, after which we recovered the catalyst by centrifugation. The powder obtained was dissolved in acetonitrile and employed in H_2_ photosynthesis as in the experimental conditions of Figure 7a. Finally, we recovered by centrifugation RuO_2_ from the organic media and we re-employed in H_2_ photoproduction in water. In Figure 7b, we reported the H_2_ produced in the photocatalytic cycles which confirm the notable activities in the different solvents and remarkable recyclability of the catalyst, indicating the potential of extremely high turnover number for this HEC.

## 3 Conclusions

In conclusion, we have described the application of commercial RuO_2_ Ns as a HEC in photocatalytic H_2_ generation. The catalyst has been employed in a classic three-component system based on the [Ru(bpy)_3_]^2+^/MV^2+^ photoinduced electron transfer and by using different electron sources to close the photocatalytic cycle. In water, we obtained a hydrogen evolution rate of 0.137 mol h^–1^ g^–1^, one of the highest reported in the literature, with EDTA·2Na, and we achieved almost half of this rate using l-cysteine. This natural amino acid is avoided with a widely used metal catalyst because the thiol moiety inhibits the catalytic activity at the surface. Furthermore, we reported the impressive activity of RuO_2_ in organic media, in particular, in acetonitrile, comparable to that obtained in water. Moreover, the possibility to recover the catalyst by centrifugation allowed several HEC cycles from different solvents without any decrease in the activity. These findings are a significant advance compared to the classical PtNps, which have several limitations in such experimental conditions.

## 4 Experimental Section

### 4.1 Materials

1,1′-Dimethyl-4,4′-bipyridinium dichloride (MVCl_2_, >98%), disodium ethylenediaminetetraacetate dihydrate (EDTA·2Na 2H_2_O, >99%), l-cysteine (>99%), triphenylphosphine (TPP, >99%), chloroplatinic acid (H_2_PtCl_6_, 99.995% trace metal basis), and poly(vinyl alcohol) (PVA, MW ≈ 130 000, >99% hydrolyzed) were purchased from Merck and used with no further purification. Tris(2,2′-bipyridyl)ruthenium(II) chloride hexahydrate ([Ru(bpy)_3_]Cl_2_ 6H_2_O, 99.95%) was purchased from Merck and re-crystallized from methanol. Anhydrous ruthenium(IV) oxide (RuO_2_, ≥99.9%) was purchased from STREM Chemicals. N_2_ used for purging (filtered on Drierite, 99.9995% purity) was supplied by Nippon Gases. Type 1 ultrapure water was obtained with an Elga PURELAB Classic UV apparatus; all other spectrophotometric grade solvents were supplied by Merck.

### 4.2 Methods

UV/vis absorption spectra were recorded on a PerkinElmer λ45 or an Agilent Cary 300 double-beam spectrophotometer using a quartz gastight cuvette with 1 cm path length; emission spectra were recorded on a PerkinElmer LS55 spectrofluorometer equipped with a Hamamatsu R928 photomultiplier tube or an Edinburgh Instruments FS5 spectrofluorometer equipped with a Hamamatsu R13456 photomultiplier tube.

X-ray diffraction (XRD) scans were carried out with a PANalytical X’Pert PRO diffractometer in the Bragg–Brentano geometry equipped with a Cu K source (λ = 1.5418 Å, 40 mA, 40 kV), and data were collected with a fast X’Celerator detector. High-angle annular dark-field scanning transmission electron microscopy (HAADF-STEM) was performed on a FEI Tecnai F20 equipped with a Schottky emitter operating at 200 kV. The determination of the hydrodynamic diameter distributions of the centrifugated Ns was carried out by DLS measurements with a Malvern Nano ZS instrument with a 633 nm laser diode; the samples were housed in quartz cuvettes of 1 cm optical path length.

### 4.3 H_2_ Production Measurements and Quantification

For exact quantification of evolved H_2_, 10 mL of the reaction mixture ([Ru(bpy)_3_]^2+^ 25 μM, MV^2+^ 5 mM, ES 0.1 M, and 200 μg of HEC) was placed in a cylindrical quartz cuvette with 5 cm path length connected to an SRI 8610C gas chromatograph equipped with a thermal conductivity detector (TCD) and a flame ionization detector (FID). The separation was performed under isothermal conditions (*T*_column_ = 50 °C) using argon as a carrier (5 mL/min, controlled by a mass flow meter). Gas was continually flowed through the cell in the dark, while the solution was stirred, and gas samples were automatically taken every 15 min for measurement to monitor the purging process. After this, irradiation, carried out with a 460 nm high-power LED (LED Engin LuxiGen LZ1-10B202-0000 operating at 600 mA, see the Supporting Information for spectral irradiance) at 5 cm distance from the quartz window (irradiated surface *S* = 2.0 cm^2^), was started, and the evolved H_2_ was monitored by injecting 1 mL of the sample every 15 min. During the same measurement, eventual CO_2_ evolution is also detected. Both detectors were calibrated by injecting 1 mL of standard gas mixtures of H_2_ and CO_2_ (5, 20, 100, and 1000 ppm of each component) supplied by Air Liquide.

## Supporting Information Available

Detailed experimental description, materials synthesis and characterization, and H_2_ production measurements and quantification (PDF)
