# Supplementary material for: RuO2 Nanostructure as an Efficient and Versatile Catalyst for H2 Photosynthesis
Source: ACS Appl Energy Mater. 2023 May 24;6(11):6243–50. doi: 10.1021/acsaem.3c00764 (PMC10265652; doi:10.1021/acsaem.3c00764)
Supplement: Supplementary file 1 — ae3c00764_si_001.pdf [file ae3c00764_si_001.pdf]

Supporting information for

RuO_2 nanostructure as efficient and versatile catalyst for H_2 photosynthesis

Alberto Bianco,[1] Alessandro Gradone,[2] Vittorio Morandi[2] and Giacomo Bergamini[1]

[1] *Department of Chemistry ''Giacomo Ciamician'', University of Bologna, Via Selmi, 2, 40126, Bologna, Italy.*

[2] *CNR Institute for microelectronics and microsystems, Via Gobetti 101, 40129, Bologna, Italy.*

Corresponding author's e-mail address: giacomo.bergamini@unibo.it

Time evolution of research papers about RuO_2 for hydrogen generation

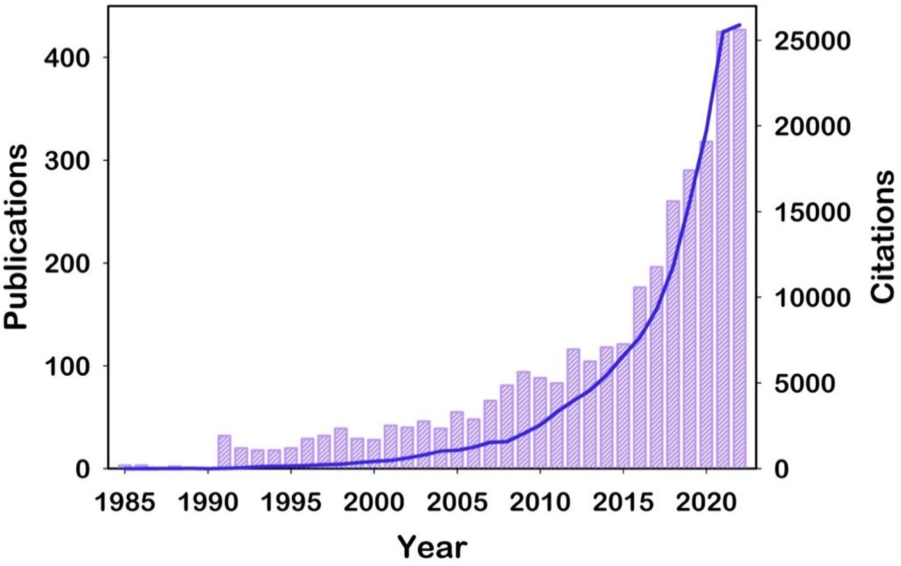

Figure S1. Time evolution of papers and citation about ('RuO_2' OR 'Ruthenium oxide') AND ('H_2' OR 'Hydrogen') from 1985 to 2022, research on Web of Science Core Collection (February 2023, the 1st).

RuO_2 characterization

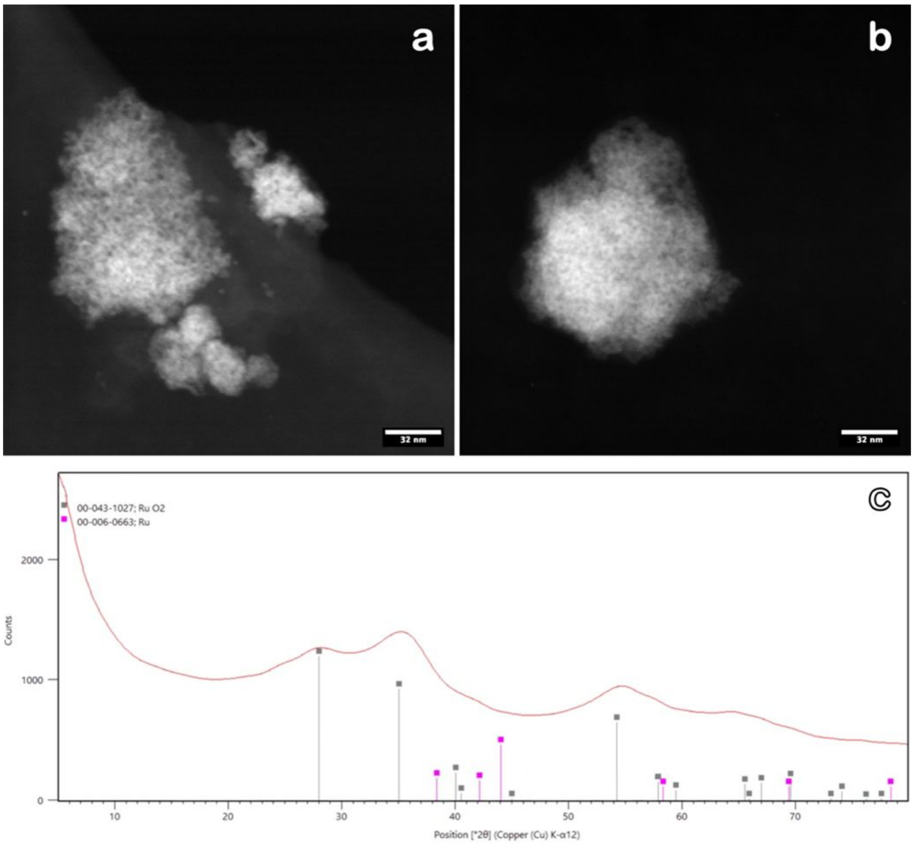

Figure S2. STEM images of RuO_2 Ns before (panel a) and after (panel b) H_2 photosynthesis (scale = 32 nm). XRD pattern of ball milled RuO_2 powder, characteristic peaks of RuO_2 and metallic Ru are shown as grey and pink dots respectively (panel c)**.**

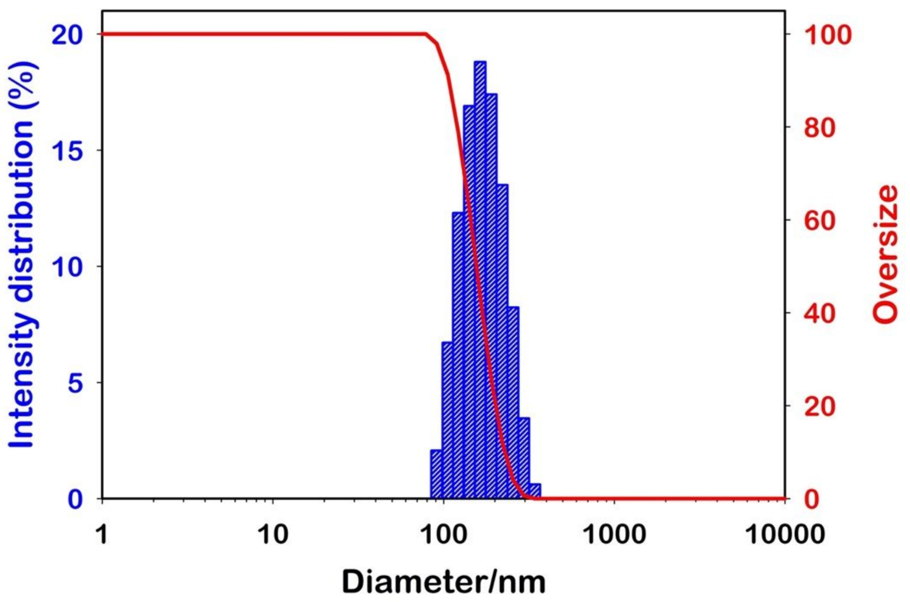

Figure S3. Intensity distribution (blue columns) obtained with dynamic light scattering analysis of centrifugated RuO_2 Ns in water (0.04% m/v). Oversize (red line) defined as the cumulative percent frequency larger than the mean diameter of each size fraction.

PtNps@PVA synthesis and characterization

PVA-stabilized platinum nanoparticles (PtNPs@PVA) were synthetized according to literature procedure[1] and characterized by STEM. The distributions of the particles dimension and morphology are pretty homogeneous. The nanoparticles diameter is around 1 - 2 nm.

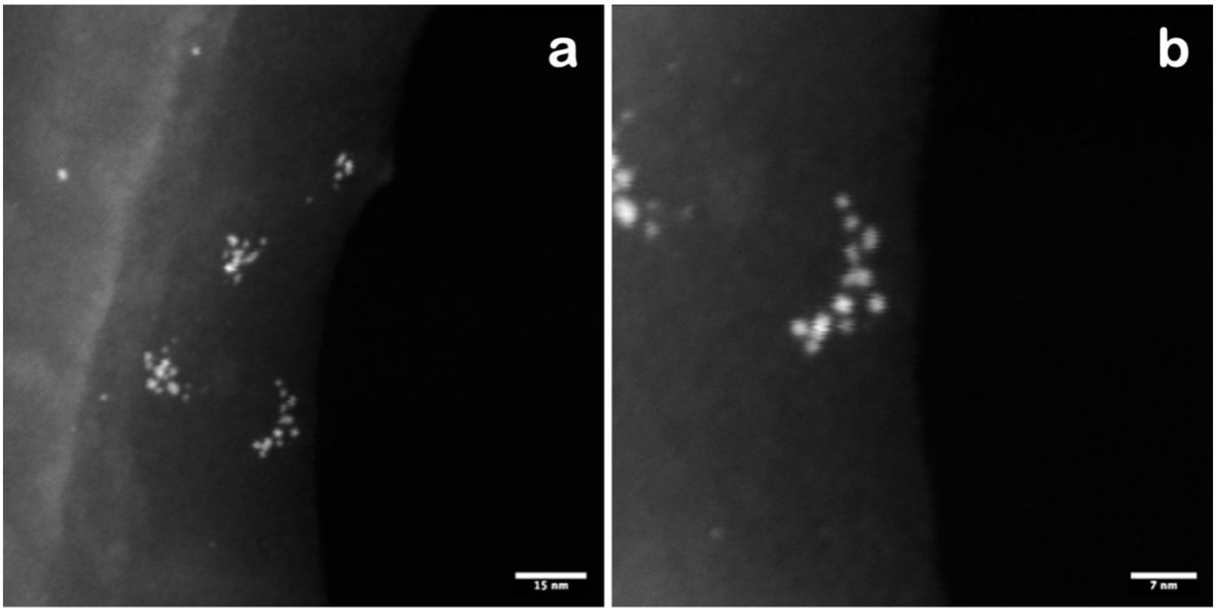

Figure S4. STEM images of PtNps@PVA at different magnifications (panel a scale = 15 nm; panel b scale = 7 nm).

To compare the masses of the Pt and Ru-based catalysts, we considered the RuO_2 mass used in the 10 mL experiment (0.2 mg, 1.5 μmol of RuO_2 MW = 133.07 $g{\cdot}mol^{-1}$) and multiplying it by 0.76, the

mass fraction of the metal respect to the whole oxide, we obtained 1.14 µmol of ruthenium. Then, the same molar amount of Pt (0.22 mg) was employed in the comparative experiment.

Quenching experiments

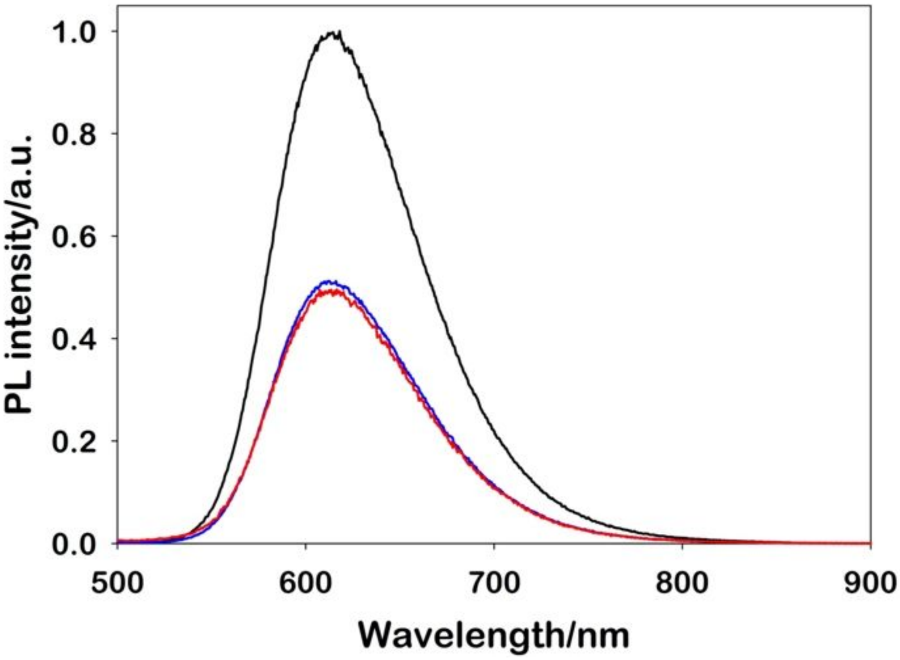

Figure S5. Uncorrected emission spectra of $[Ru(bpy)_3]^{2+}$ in deaerated aqueous solution with no quencher (black line), upon 5 mM MV^{2+} addition (blue line) and subsequent 0.1 M EDTA addition (red line) (λ_{exc}= 460 nm). The quenching of the luminescence in the reaction conditions (estimated by integral ratio) is 50%.

RuO_2 recovery

The catalyst was recovered with the following procedure: the mixture was placed in 15 mL conical tube and centrifugated for 10 minutes at 10 000 rpm (12630 G), then the supernatant was removed and the solid was redispersed in 10 mL of clean solvent (H_2O or MeCN, depending on the subsequent irradiation condition). This procedure was repeated three times, obtaining a clean supernatant and a black solid residual. The latter was then redispersed in 10 mL of reaction mixture.

H_2 production optimization

Rapid pre-screening of different conditions for H_2 evolution were performed using a home-made instrument composed of a 3D-printed gas-tight cell, a *MQ-8* gas sensor, an *Arduino Uno* microcontroller and a PC running *Microsoft Excel* and *Parallax PLX-DAQ*. For these measurements 2 mL of hydrogen evolving mixture ($[Ru(bpy)_3]^{2+}$ 25µM, MV^{2+} 5mM, ES 0.1M and HEC) were placed in a quartz cuvette with 1 cm path length inside the holder, irradiated with a 460 nm high-power LED (LED Engin LuxiGen™ LZ1-10B202-0000 operating at 600mA) at 5 cm distance from the quartz window for 30 minutes under vigorous stirring, and recording

the total H_2 level every 10 seconds. Since is not possible to perform an absolute calibration of this sensor, the readout measure (in ppm) is not an exact quantification of the produced H_2 but they are definitely reproducible and reliable for comparison.

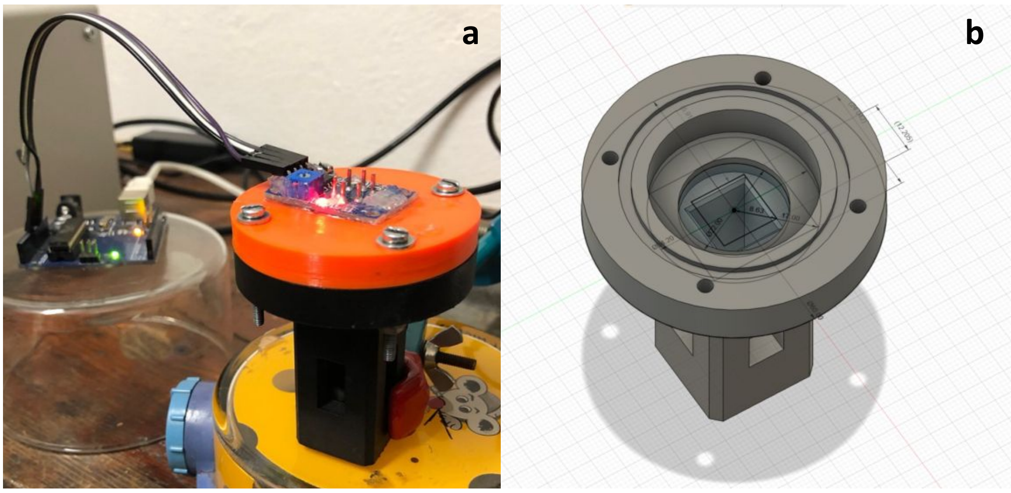

Figure S6. 3D-printed gas-tight cell with MQ-8 gas sensor and *Arduino Uno* board (panel a). Fusion360 cad project of the 3D-printed gas-tight cell (panel b).

H_2 production measurements and quantification

For exact quantification of evolved H_2, 10 mL of reaction mixture ($[Ru(bpy)_3]^{2+}$ 25 µM, MV^{2+} 5 mM, ES 0.1 M and 200 µg of HEC) were placed in a cylindrical quartz cuvette with 5 cm path length connected to an *SRI 8610C* gas-chromatograph equipped with a *Thermal Conductivity Detector* (TCD) and a *Flame Ionization Detector* (FID). The separation was performed under isothermal conditions (T_{Column} = 50°C) using argon as a carrier (5 mL/min, controlled by a *mass flow meter*). Gas was continually flowed through the cell in the dark while the solution was stirred and gas samples were automatically taken every 15 minutes for measurement to monitor the purging process.

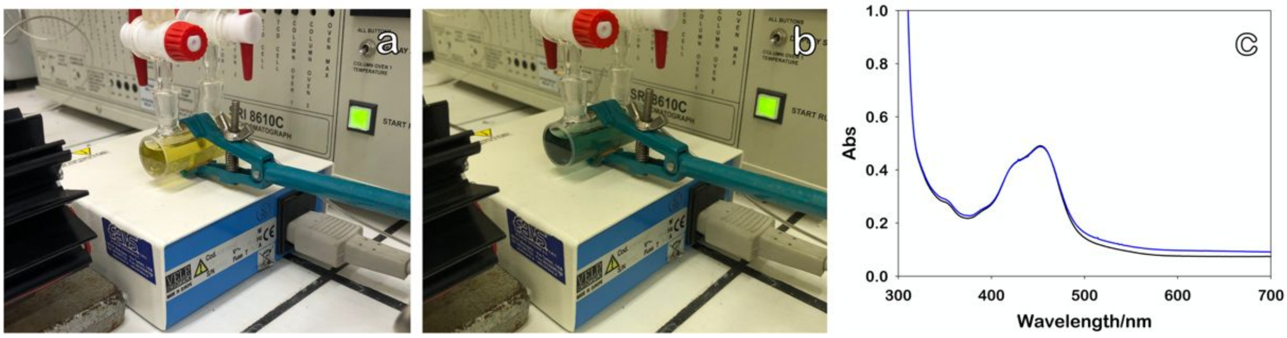

Figure S7. Reaction mixture before (panel a) and during (panel b) H_2 photosynthesis using RuO_2 in acetonitrile. Panel c shows absorption spectra before (black line) and after one hour irradiation (blue line).

After this, the irradiation, carried out with a 460 nm high-power LED (LED Engin LuxiGen™ LZ1-10B202-0000 operating at 600mA) at 5 cm distance from the quartz window (Irradiated surface $S =$

$2.0\ cm^2$), was started and the evolved H_2 was monitored injecting 1 mL of sample every 15 minutes. During the same measurement also eventual CO_2 evolution is detected. Both detectors were calibrated injecting 1 mL of standard gas mixtures of H_2 and CO_2 (5, 20, 100 and 1000 ppm of each component) supplied by *Air Liquide*.

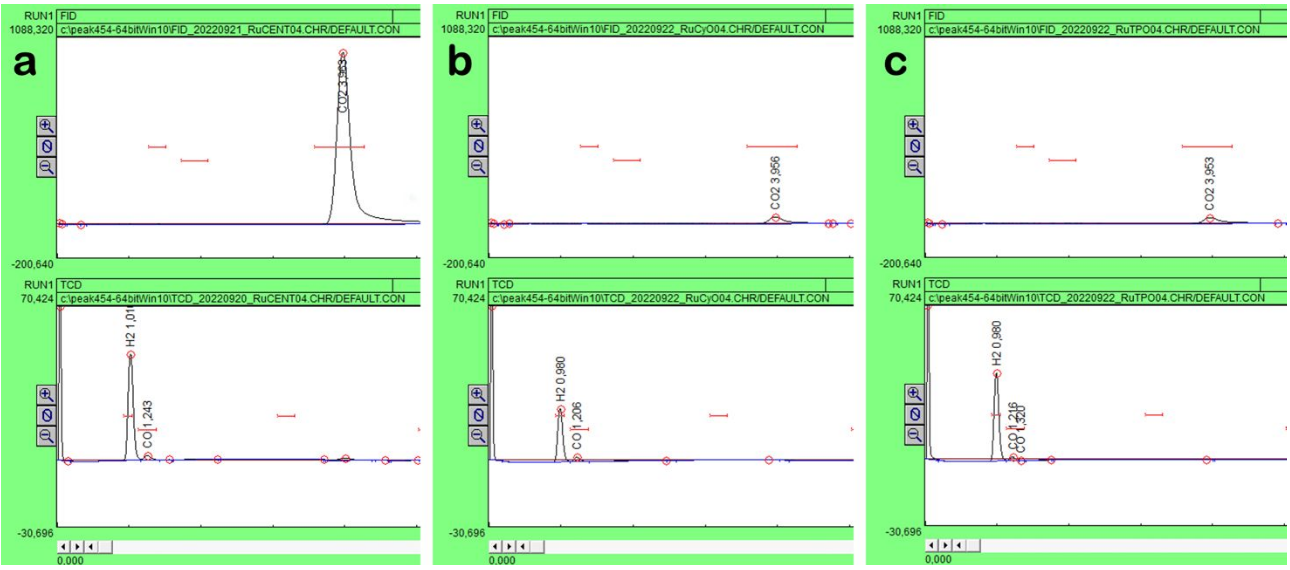

Figure S8. Comparison of GC analysis using EDTA (a) or L-cysteine (b) in water and TPP in acetonitrile (c) as ES. The CO_2 peak at 3.95 min in panel a comes from the oxidation of EDTA, in panel b and c is ascribable to the residual gas dissolved in the solution.

The GC measurement results with an area for the H_2 peak which is converted in H_2 concentration (in ppm) in 1 mL of sample (loop volume) using the aforesaid calibration.

$$A_{peak} \propto [H_2]_{ppm}$$

Considering the flow of the carrier gas (5 mL/min), it is possible to correlate the 1 mL sample to 12 seconds of H_2 evolution so, it is possible to convert this result in *Hydrogen Evolution Rate* (in mL/s) described as:

$$H_{2\ evolution\ rate} = \frac{[H_2]_{ppm}}{12 \cdot 10^6}\left[\frac{mL}{s}\right] = \frac{[H_2]_{ppm}}{12 \cdot 10^9}\left[\frac{L}{s}\right]$$

Then, considering $T = 298.15\ K$ and $p = 1\ atm$, it is possible to convert the volume of evolved gas in number of molecules using the following formula:

$$H_{2\ evolution\ rate} = \frac{[H_2]_{ppm} \cdot N_A}{12 \cdot 10^9 \cdot 24{,}45} \left[\frac{molecules}{s}\right]$$

In which N_A is the *Avogadro constant* and $24{,}45$ are the litres occupied by a mole of perfect gas in the conditions described above.

Hydrogen evolution rates can also be reported to the mass of catalyst in the following way:

$$H_{2\ evolution\ rate} = \frac{[H_2]_{ppm} \cdot 3600}{12 \cdot 10^9 \cdot 24{,}45 \cdot m_{HEC}} \left[\frac{mol}{h \cdot g}\right]$$

Were 3600 are the seconds in one hour and m_{HEC}represents the mass of catalyst in grams.

The calculated hydrogen evolution rates (obtained by fitting linearly the experimental data) are reported in the table below:

**Irradiation condition**						
**$[Ru(bpy)_3]^{2+}$ 30 µM**	**MV^{2+} 5 mM**	**ES 0.1 M**	**Solvent**	**HEC**	**HER molecules · s^{-1}**	**HER mol · h^{-1} · g^{-1}**
YES	YES	EDTA·2Na	H_2O, pH = 4.9	RuO_2	$4.6 \cdot 10^{15}$	0.137
YES	YES	EDTA·2Na	H_2O, pH = 4.9	PtNps	$4.6 \cdot 10^{15}$	0.125
YES	YES	L-cysteine	H_2O, pH = 4.9	RuO_2	$1.9 \cdot 10^{15}$	0.057
YES	YES	L-cysteine	H_2O, pH = 4.9	PtNps	$0.2 \cdot 10^{15}$	0.005
YES	YES	TPP	MeCN, HCl 10 mM	RuO_2	$4.6 \cdot 10^{15}$	0.137
YES	YES	TPP	MeCN, HCl 10 mM	PtNps	$0.3 \cdot 10^{15}$	0.008
NO	YES	EDTA·2Na	H_2O, pH = 4.9	RuO_2	0	0
NO	YES	EDTA·2Na	H_2O, pH = 4.9	PtNps	0	0
YES	NO	EDTA·2Na	H_2O, pH = 4.9	RuO_2	0	0
YES	NO	EDTA·2Na	H_2O, pH = 4.9	PtNps	0	0
YES	YES	NONE	H_2O, pH = 4.9	RuO_2	0	0
YES	YES	NONE	H_2O, pH = 4.9	PtNps	0	0
YES	YES	EDTA·2Na	H_2O, pH = 4.9	NONE	0	0

Table S1. Measured hydrogen evolution rates obtained using the optimized amount of RuO_2 (0.20 mg) or PtNps (0.22 mg) as HEC in different irradiation conditions.

Apparent quantum efficiency for H_2 photosynthesis

The most straightforward measurement to estimate the incident photons is absolute spectral irradiance $E_{e,\lambda}$, which is the radiant flux received by a surface per unit area per wavelength $\left[\frac{W}{m^2 \cdot nm}\right]$.

To measure it we used a diode array detector spectrometer *Avantes AvaSpec 2048* equipped with an optical fiber (200 µm diameter, 1 m length) and a cosine corrector, calibrated with an irradiance standard (*OL245C* supplied by *Optronic Laboratories*).

Once obtained the spectral irradiance of the source in the same geometry of the experiment, it is possible to multiply this for the irradiated surface S, obtaining the spectral flux $\Phi_{e,\lambda}$, which is the radiant energy received per unit time per wavelength $\left[\frac{W}{nm}\right]$:

$$\Phi_{e,\lambda} = E_{e,\lambda} \cdot S$$

To convert the spectral flux in photons per seconds at each wavelength it is necessary to divide it by the energy of the photons at the specific wavelength $\left[\frac{n^\circ\ Photons}{s \cdot nm}\right]$:

$$\frac{Incident\ photons_\lambda}{s} = \frac{\Phi_{e,\lambda}}{h \cdot \nu_\lambda} = \frac{E_{e,\lambda} \cdot S}{h \cdot \nu_\lambda} = \frac{E_{e,\lambda} \cdot S \cdot \lambda}{h \cdot c}$$

Where h is the Planck's constant $(6.62607015 \cdot 10^{-34}\ J \cdot s)$, ν_λ is the frequency associated to the λ wavelength and c is the speed of light in vacuum $(299\ 792\ 458\ {}^{m}/_{s})$. Therefore, the total number of incident photons per second $\left[\frac{n^\circ\ Photons}{s}\right]$ is given by the following formula:

$$\frac{Incident\ photons}{s} = \frac{S}{h \cdot c} \cdot \int_{\lambda_1}^{\lambda_2} E_{e,\lambda} \cdot \lambda d\lambda$$

The integral is evaluated over the entire gaussian profile of the source, obtaining $1.35 \cdot 10^{17}$ ${}^{Incident\ photons}/_{s}$ at photoreactor' surface $(S = 2.0\ cm^2)$.

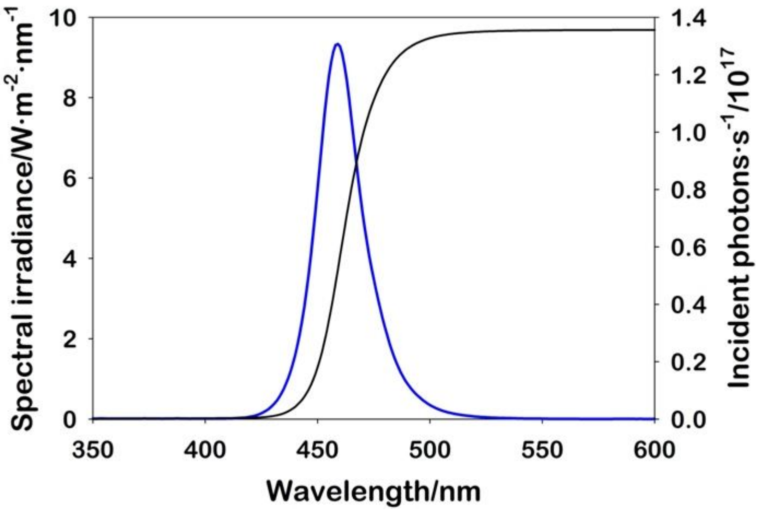

Figure S9. Spectral irradiance of LED Engin LuxiGen™ LZ1-10B202-0000 operating at 600mA at 5 cm distance (blue line) and number of photons integration over the 350 – 600 nm spectral range (black line).

Since the bandwidth of the light source used in this work is very narrow (448 - 471 nm *FWHM*, 0.14 eV) and the absorbed light fraction in that range is greater than 99 % ($Abs \geq 2$) it is possible to calculate the *Apparent Quantum Efficiency* (AQE), defined as:

$$AQE_{PTH} = \frac{2 \cdot H_2 molecules}{Incident\ photons}$$

Considering the number of incident photons measured above and the data reported in table S1, it is possible to obtain the following AQE values:

Irradiation condition					AQE
$[Ru(bpy)_3]^{2+}$ 30 µM	MV^{2+} 5 mM	ES 0.1 M	Solvent	HEC	
YES	YES	EDTA·2Na	H_2O, pH = 4.9	RuO_2	6.8 %
YES	YES	EDTA·2Na	H_2O, pH = 4.9	PtNps	6.8 %
YES	YES	L-cysteine	H_2O, pH = 4.9	RuO_2	2.8 %
YES	YES	L-cysteine	H_2O, pH = 4.9	PtNps	0.3 %
YES	YES	TPP	MeCN, HCl 10 mM	RuO_2	6.8 %
YES	YES	TPP	MeCN, HCl 10 mM	PtNps	0.4 %

Table S2. Apparent quantum yield (photon-to-hydrogen) obtained using the optimized amount of RuO_2 (0.20 mg) or PtNps (0.22 mg) as HEC in different irradiation conditions.

L-Cysteine oxidation product

To verify the disulphide formation using L-Cysteine as electron donor the following experiment was carried out: 5.0 mL of of $[Ru(bpy)_3]^{2+}$ (30.0 µM), MV^{2+} (5.0 mM), L-cysteine (0.1 M) and RuO_2 (0.1 mg) pH 4.9 water solution were divided in two equal parts (2.5 mL); both were deaerated by bubbling Argon. The first sample was irradiated at 460 nm for 4 hours while the second was kept in the dark.
After irradiation both samples were centrifugated to remove the catalyst and the obtained solutions were concentrated by rotary evaporation and dried under vacuum, obtaining a faint yellow solid for both samples. These two were analysed through *Attenuated Total Reflection Fourier Transform InfraRed spectroscopy* (ATR-FTIR) with a Bruker ALPHA II spectrometer.

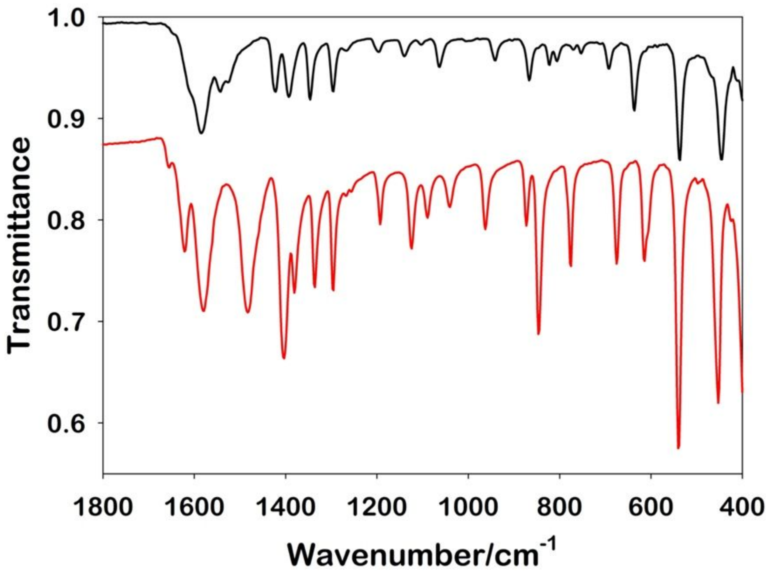

Figure S10. IR spectra of dark (black line) and irradiated (red line, shifted in transmittance by subtracting 0.1) solid samples.

The figure S10 shows a perfect match with already reported L-Cysteine spectra for the dark sample and with L-Cystine for the irradiated one.[2,3]

References

(1) Toshima, N.; Kuriyama, M.; Yamada, Y.; Hirai, H. Colloidal Platinum Catalyst For Light-Induced Hydrogen Evolution From Water. A Particle Size Effect. *Chem. Lett.* **1981**, *10* (6), 793–796. https://doi.org/10.1246/cl.1981.793.

(2) Bastian, E. J. J.; Martin, R. B. Disulfide Vibrational Spectra in the Sulfur-Sulfur and Carbon-Sulfur Stretching Region. *J. Phys. Chem.* **1973**, *77* (9), 1129–1133. https://doi.org/10.1021/j100628a010.

(3) Koleva, B.; Spiteller, M.; Kolev, T. Polarized Spectroscopic Elucidation of N-Acetyl-l-Cysteine, l-Cysteine, l-Cystine, l-Ascorbic Acid and a Tool for Their Determination in Solid Mixtures. *Amino Acids* **2010**, *38* (1), 295–304. https://doi.org/10.1007/s00726-009-0250-y.
